# Research collaboration in health management research communities

**DOI:** 10.1186/1472-6947-13-52

**Published:** 2013-04-23

**Authors:** Chichen Zhang, Qi Yu, Qinghua Fan, Zhiguang Duan

**Affiliations:** 1 School of Public Health, Shanxi Medical University, South Xinjian Road, Taiyuan, China; 2 Department of Information Management, Shanxi Medical University, South Xinjian Road, Taiyuan, China; 3 Division of International Cooperation and Exchange, Shanxi Medical University, South Xinjian Road, Taiyuan, China; 4 School of Public Health, Shanxi Medical University, South Xinjian Road, Taiyuan, China

**Keywords:** Health management, Co-authorship, Network, Collaboration

## Abstract

**Background:**

This study uses scientometrics methodology to reveal the status quo and emerging issues of collaboration in health management.

**Methods:**

We searched all the articles with the keyword “health management” in the period 1999–2011 in Web of Knowledge, then 3067 articles were found. Methods such as Social network analysis (SNA), co-authorship, co-word analysis were used in this study.

**Results:**

Analysis of the past 13 years of research in the field of health management indicates that, whether the production of scientific research, or authors, institutions and scientific research collaboration at the national level, collaboration behavior has been growing steadily across all collaboration types. However, the international scientific research cooperation about health management study between countries needs to be further encouraged. 17 researchers can be seen as the academic leaders in this field. 37 research institutions play a vital role in the information dissemination and resources control in health management. The component analysis found that 22 research groups can be regarded as the backbone in this field. The 8 institution groups consisting of 33 institutions form the core of this field. USA, UK and Australia lie in the center by cohesive subgroup analysis; Based on keywords analysis, 44 keywords with high frequency such as care, disease, system and model were involved in the health management field.

**Conclusions:**

This study demonstrates that although it is growing steadily, collaboration behavior about health management study needs to be enhanced, especially between different institutions or countries/regions, which would promote the progress and internationalization of health management. Besides, researchers should pay attention to the cooperation of representative scholars and institutions, as well as the hot areas of research, because their experience would help us promote the research development of our nation.

## Background

The idea and practice of health management originated from America in 1950s, and then sprung up as an emerging subject in UK, Germany, France, Japan etc. In the 21st century, health management spread in the developing countries and was applied in government, business, medical institutions and the insurance industry. Now it has become a prospective health service model for many countries to improve their national health level and promote the society’s sustainable development. Along with the increasingly popularity of health management, research collaboration in this field has also increased. Research collaboration is becoming an important way of improving health management by extensive cooperation, which makes resources sharing and knowledge stocking possible. However, the co-authorship analysis in this field is seldom reported. Thus, the status quo of international collaboration in health management was revealed by scientometric methodology in this study.

## Literature review

It has been argued that co-authorship do not provide the entire view of the process of collaboration, however it is still advantageous for collaboration analysis through co-authorship as it is inexpensive and practical [1-4]. Co-authorship can be analyzed at three levels (authors, institutions and countries/regions), such as the analysis of different countries/regions, institutions and authors for a certain time. It is a way to reveal the interrelationships of the domain, the intensity of these relations [5,6]. Also, all sorts of methods are applied to this field, including the frequently used Bibliometric techniques and social network analysis, as well as some new methods. Moreover, Zaida Chinchilla-Rodríguez had used blockmodeling to study the internal structure of co-authorship networks in the micro-level in 2012 [7].

Publications with more than one author have been on the rise, with many studies showing this trend [8-11]. However, these trends are not uniform, and must be contextualized by domain, country conditions and field of study [12-17]. Research in this respect has shown that there is a rise in institutional collaboration [18], but with the full caveats that this varies by discipline [19]. Especially in the biomedical fields, it tends to have high degrees of collaboration between institutions domestically, but not internationally [20].

The increase in international collaboration is not only a trend of the 21st century, but one that has been noted in scientometric studies for over a decade [21-26]. However, very few studies examined the collaboration activities in Health Management research field across multiple collaboration types [27-31]. This study intends to address this issue.

## Research questions

We intended to reveal current status of the collaboration activities and research topics in the Health Management field by using the method of co-authorship and co-word analysis so as to provide scientific evidence on research collaboration and suggestions for policymakers to establish a more efficient system for guiding and funding the Health Management research in the future.

Research Question 1:

What is the research collaboration trend in Health Management research?

Research Question 2:

Who/which are the most collaborative authors, institutions and countries/regions in Health Management research?

Research Question 3:

What are the research topics in Health Management research?

## Data and methods

### Date collection

The documents which contain the word “Health Management” in their title, abstract or keywords were collected from the scientific literature database “Web of Knowledge”. The scope was limited to the years 1999 through 2011. All documents regardless of type (e.g. article, meeting abstract, proceedings paper, review, editorial material, book review, letter, note, etc.) were processed. All documents from the Science Citation Index Expanded (SCI-Expanded), Social Sciences Citation Index (SSCI), Arts & Humanities Citation Index (A&HCI), Conference Proceedings Citation Index-Science (CPCI-S), and Conference Proceedings Citation Index-Social Science & Humanities (CPCI-SSH) were taken into account. The query yielded 3067 records, each of which has author names, affiliations, titles, sources, abstracts, total citations, key words and cited references.

### Data Refinement

Articles coauthored by authors from more than one institution were classified as multi-institutional collaboration. A paper coauthored by authors from different countries/regions was considered a multi-national paper.

The names of authors and institutions have been normalized manually. For example, Zhao Y from Shanghai Univ was labeled “Zhao Y 1”, while Zhao Y from Sch Management Beihang Univ was labeled “Zhao Y 2”. Different variations of institution’s name were assigned to one name.

Keywords Plus (Web of Knowledge supplied keywords in capital letters) is used in this paper.

## Methods

In our previous studies, we revealed the collaboration activities in the oncology research field and cardiology and cardiovasology research field by means of coauthorship analysis, social network analysis and keyword analysis. We believe that these studies can provide suggestions for policy-maker in medical research management.

### Bibliometrics

Bibliometrics is a quantitative analysis method by processing the literatures’ characteristics and using mathematics and statistics methods to describe, evaluate and predict the status and future of science and technology.

### Social network analysis

Social network is a network of individuals’ communication including nodes and ties, especially for gaining one’s specific ends. The node represents the individual or institution in the network, while the tie represents the content or way of communication [32,33]. Social network analysis (SNA) is the methodical analysis of social networks. Social network analysis views social relationships in terms of network theory, consisting of nodes (representing individual actors within the network) and ties (which represent relationships between the individuals, such as friendship, kinship, organizational position, sexual relationships, etc.) [34-37].

Pajek, a visualization toolkit for large-scale networks, was applied to map the collaboration. The node size in the graph is proportional to number of productions by authors, institutions or countries/regions, and the thickness of the lines represents the number of co-published papers.

### Centrality

Centrality is an important index to analyze the network. Whether the individual or institution lies in the center of social network will determine its influence on the network and its speed to gain information. Centrality measure includes degree centrality, closeness centrality, and betweenness centrality.

Degree Centrality of is defined as the number of ties that a node has. Degree Centrality represents the simplest notion of Centrality since it is just the number of neighbors of a node in the network.

The Closeness Centrality of a node is the number of others nodes divided by the sum of all geodesic distances between the node and all others, where larger distances yield lower Closeness Centrality scores. The closer a node is to all other nodes, the easier information may reach it, the higher its Centrality.

Betweenness Centrality rests on the idea that a node is more central if he is more important as an intermediary in the network. The Betweenness Centrality of a node is the proportion of all geodesics between pairs of other nodes that include the node.

The three centrality metrics can help us identify the “important” persons or organizations in the network.

### N-cliques and M-core

N-cliques insists that every member or a sub-group have a direct tie with each and every other member. M-core is a cohesive subgroup which meets the requirements that all line value in the subgroup are no less than M.

### Keyword co-occurrence analysis

Methods such as co-citation analysis, bibliographic coupling analysis and keyword analysis can be used to reveal the hot research topics. Co-citation analysis, bibliographic coupling analysis are two citation-based approaches. As many of our records have no citations, we chose keyword analysis in our study. If two keywords co-occur in many articles, it implies the close links between the topics to which they refer. Therefore, the analysis of the keyword co-occur frequency could reflect the relationship of the subjects. The keyword co-occurrence has been used in many studies to reveal research hot topics of some specific field or discipline [38-42]. In this study, keyword co-occurrence was used to provide an immediate picture of research collaboration topics in health management field.

## Results

### The trends of scientific production in the area of health management

**Figure 1:**
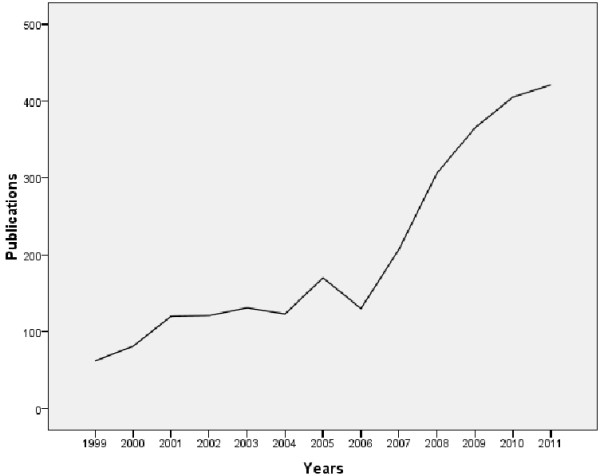
Evolution of publications in health management research from 1999 to 2011.

From 1999 to 2011, the total amount of research papers in the field of health management has a significant growth (Figure 1). Figure 1 shows the total number of papers published annually. Overall, in 13 years the number of published articles increased by nearly six times from 62 in 1999 to 421 in 2011.

### The analysis of cooperation trends

#### The trend of co-author

**Figure 2:**
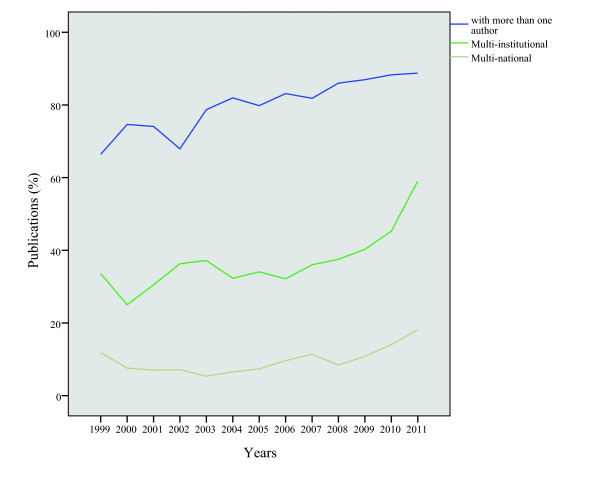
Percentage of multi-entity publications in health management research, 1999–2011.

**Figure 3:**
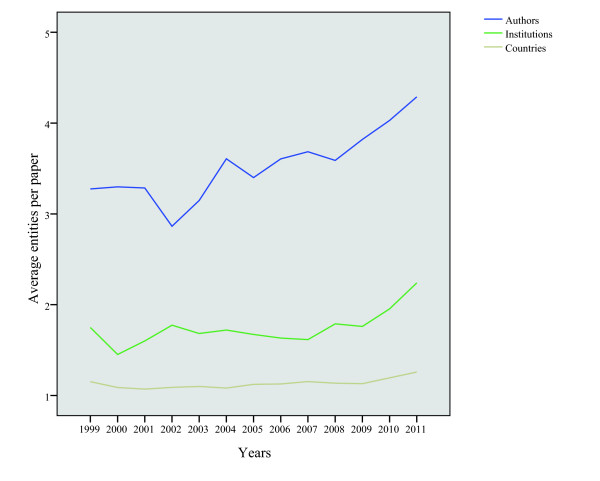
Average numbers of different entities per paper in health management research, 1999–2011.

Between 1999 and 2011, the collaboration among the staff of health management has increased significantly. Figure 2 displays the percentage of writers coauthored papers, institutions coauthored papers and nationality coauthored papers. Figure 3 reveals the change of the average article number for an author, institution or country. The ratio of coauthored papers increased from 66% in 1999 to 89% in 2011. The ratio of institutions coauthored papers and national coauthored papers showed a similar trend of growth. However, the ratio of papers coauthored by writers is significantly higher than the other two resources. The quantity of the average article number for an author went up from 3.27 in 1999 to 4.29 in 2011.

#### The trend of institutional co-authorship

As mentioned above, in the field of health management research, the ratio of papers coauthored by institution grew from 34% in 1999 to 59% in 2011. Meanwhile, the average article number for institution climbed from 1.75 in 1999 to 2.24 in 2011.

#### The trend of national co-authorship

As shown in figure 2, the ratio of national coauthored papers in the field of health management research increased from 12% in 1999 to 18% in 2011. Figure 3 shows, most of the papers are the achievements of cooperation within a country, and the average article number for a country increased from 1.15 in 1999 to 1.26 in 2011, which increased slowly compared with that of researchers coauthored papers and institutions coauthored papers.

### Collaborations among researchers

#### Co-author network

The co-authorship network in this study contains 9447 nodes (researchers), 22666 lines (co-author frequency). The maximum co-author frequency is 8 and the network density is 0.000508.

#### Centrality analysis

There are 17 authors who ranked in top 100 of all the three centralities in the co-authorship network, measured by calculating the degree, closeness and betweenness centrality (See Table 1).

**Table 1:** Top 17 authors with high centralities

**Rank**	**Authors**	**Degree**	**Closeness**	**Betweeness**
1	"O'Toole, T1"	57	0.006160686	1.38332E-05
2	"Ascher, MS"	51	0.005938011	7.10807E-06
3	"Tonat, K2"	46	0.005764384	9.00965E-06
4	"Osterholm, MT2"	46	0.005764384	5.15801E-06
5	"Perl, TM2"	45	0.005730871	4.96446E-06
6	"Hauer, J2"	40	0.005568982	3.76404E-06
7	"Layton, M3"	40	0.005568982	3.76404E-06
8	"Lillibridge, S"	40	0.005187946	3.13349E-06
9	"Arase, Y"	39	0.004234148	8.41763E-06
10	"Friedlander, AM"	37	0.004831911	2.43051E-05
11	"Swearingen, K"	34	0.004401286	4.85667E-05
12	"Byington, CS1"	33	0.005189133	9.19832E-05
13	"Eitzen, EM"	29	0.005243137	3.0151E-05
14	"Pecht, M1"	28	0.004568423	0.00012659
15	"Schmaljohn, AL"	28	0.004953315	1.21053E-05
16	"Peters, CJ"	28	0.004879751	6.25438E-06
17	"Roemer, MJ"	24	0.005683336	6.23491E-05

#### Cohesive subgroup analysis

The N-clique of co-authorship network in health management was shown in Table 2. The maximum clique is 31-clique and 91.66% of researchers belong to 9-clique or below. Moreover, the majority researchers belong to 2-clique, 3-clique and 4-clique, the number is 1428, 1459, 1352 respectively.

**Table 2:** The N-clique of co-authorship network in health management

**N-Clique**	**Freq**	**Freq%**	**CumFreq%**	**N-Clique**	**Freq**	**Freq%**	**CumFreq%**
0	512	5.42	5.42	11	118	1.25	94.63
1	1009	10.68	16.10	12	117	1.24	95.87
2	1428	15.12	31.22	13	84	0.89	96.76
3	1459	15.44	46.66	14	74	0.78	97.54
4	1352	14.31	60.97	15	48	0.51	98.05
5	1141	12.08	73.05	17	47	0.50	98.55
6	806	8.53	81.58	18	33	0.35	98.90
7	469	4.96	86.55	21	22	0.23	99.13
8	252	2.67	89.21	23	24	0.25	99.39
9	231	2.45	91.66	25	26	0.28	99.66
10	163	1.73	93.38	31	32	0.34	100.00

The M-core of co-authorship network in health management was shown in Table 3. The maximum core is 8-core. And the majorities are in 1-core, which contains 8181 researchers.

**Table 3:** The M-core of co-authorship network in health management

**M-Core**	**Freq**	**Freq%**	**CumFreq**	**CumFreq%**
0	512	5.42	512	5.42
1	8181	86.60	8693	92.02
2	571	6.04	9264	98.06
3	113	1.20	9377	99.26
4	37	0.39	9414	99.65
5	17	0.18	9431	99.83
6	11	0.12	9442	99.95
7	3	0.03	9445	99.98
8	2	0.02	9447	100.00

**Figure 4:**
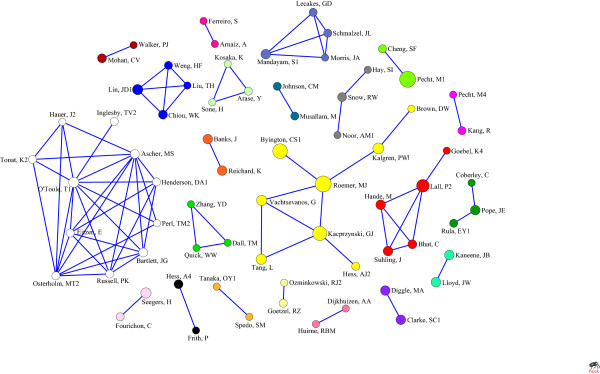
Collaboration among authors (line value>4).

By component analysis of 70 researchers who are higher than 4-core in the co-authorship, 22 groups were found (Figure 4), which means that the authors in each group co-published no less than 4 papers. And the relations among researchers within those groups are tight and stable.

### Collaborations among research institutions

#### Multi-institutional collaboration network

The institution collaboration network contains 2776 nodes (research institutions), 4461 lines. The maximum line value is 9 and the network density is 0.0011582.

#### Centrality analysis

There are 37 institutions that ranked in top 100 in the network, by calculating of degree, closeness and betweenness centrality (See Table 4).

**Table 4:** Top 37 institutions with high centralities

**Rank**	**Org**	**Degree**	**Closeness**	**Betweeness**
1	UNIV MINNESOTA	65	0.139536034	0.026331189
2	USAF	61	0.133243448	0.020084938
3	UNIV WASHINGTON	59	0.136385856	0.032965808
4	UNIV MARYLAND	55	0.123266575	0.020476832
5	CTR DIS CONTROL & PREVENT	42	0.130342946	0.011750547
6	UNIV MICHIGAN	40	0.122175917	0.015957716
7	WHO	39	0.123311505	0.016469697
8	STANFORD UNIV	38	0.13350636	0.029782294
9	COLUMBIA UNIV	38	0.130670187	0.017456569
10	JOHNS HOPKINS UNIV	34	0.128486561	0.020521669
11	UNIV ALABAMA	32	0.125066586	0.008339021
12	UNIV MELBOURNE	32	0.117405905	0.019133512
13	US DEPT HHS	32	0.129817773	0.008307802
14	UNIV MISSOURI	30	0.125484093	0.005603591
15	UNIV SO CALIF	30	0.123423975	0.004564441
16	MINIST HLTH	29	0.124217043	0.034480061
17	NORTHWESTERN UNIV	29	0.128462166	0.022695165
18	MED COLL GEORGIA	29	0.128291663	0.003952521
19	TEL AVIV UNIV	27	0.118123294	0.008396166
20	LONDON SCH HYG & TROP MED	26	0.127927818	0.014331175
21	BETH ISRAEL DEACONESS MED CTR	26	0.126209705	0.006820911
22	UNIV WISCONSIN	24	0.121386837	0.007261674
23	UNIV WESTERN AUSTRALIA	24	0.115857916	0.007170564
24	NIH	24	0.131151431	0.007359746
25	UNIV ILLINOIS	23	0.118661913	0.005502203
26	HARVARD UNIV	22	0.120264883	0.014331563
27	ALBERT EINSTEIN COLL MED	22	0.122729953	0.003980603
28	UNIV CALIF LOS ANGELES	21	0.125740612	0.003765431
29	UNIV CALIF SAN FRANCISCO	21	0.126021648	0.005312616
30	UNIV PENN	20	0.130117352	0.018659974
31	BOSTON UNIV	20	0.125717248	0.01613023
32	PENN STATE UNIV	17	0.11763043	0.00749422
33	DUKE UNIV	17	0.118516418	0.007325933
34	MCMASTER UNIV	17	0.11857873	0.003277398
35	PALO ALTO MED FDN	16	0.122774493	0.006299589
36	CHILDRENS HOSP	15	0.118745214	0.007173336
37	UNIV COLORADO	14	0.115561098	0.003574301

#### Analysis of cohesive subgroups

**Figure 5:**
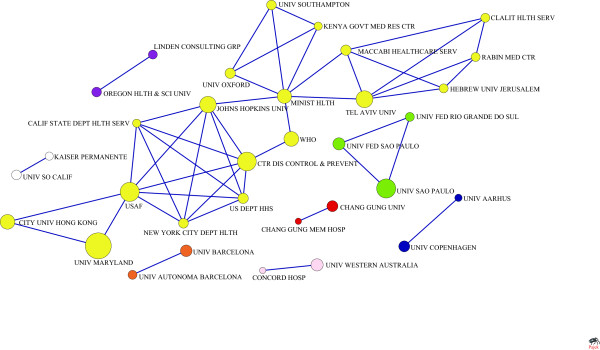
Collaboration among institutions (line value>3).

The N-clique of institution collaboration network in health management was shown in Table 5. The maximum is 16-clique. The M-core of health management institution collaboration network was shown in Table 6. The maximum is 9-core. And the majorities are in 1-core, which contains 1932 institutions. By component analysis of the 33 institutions that are in 3-core or more, 8 groups were found (Figure 5).

**Table 5:** The N-clique of institution collaboration network in health management

**Cluster**	**Freq**	**Freq%**	**Representative**
0	651	23.451	SCI MONITORING INC
1	594	21.3977	UNIV ABERDEEN
2	544	19.5965	BOEING CO
3	330	11.8876	IMPACT TECHNOL LLC
4	190	6.8444	UNIV GUELPH
5	171	6.1599	MICHIGAN STATE UNIV
6	95	3.4222	NASA
7	28	1.0086	UNIV MICHIGAN
8	49	1.7651	WHO
9	16	0.5764	LONDON SCH HYG & TROP MED
10	19	0.6844	UNIV WISCONSIN
11	33	1.1888	UNIV MARYLAND
13	25	0.9006	UNIV WASHINGTON
14	14	0.5043	USAF
16	17	0.6124	UNIV ALABAMA

**Table 6:** The M-core of health management institution collaboration network

**Cluster**	**Freq**	**Freq%**	**Representative**
0	651	23.451	SCI MONITORING INC
1	1932	69.5965	PENN STATE UNIV
2	151	5.4395	NASA
3	23	0.8285	MINIST HLTH
4	2	0.072	UNIV SOUTHAMPTON
5	11	0.3963	UNIV SAO PAULO
6	1	0.036	JOHNS HOPKINS UNIV
7	3	0.1081	PREVENT
9	2	0.072	UNIV MARYLAND

### Collaboration among countries/regions

#### Multi-national collaboration network

The countries/regions cooperation network is structured by data analysis which contains 102 nodes (countries/regions), 358 lines. The maximum frequency of co-nation is 18 and the network density is 0.0683243.

#### Analysis of cohesive subgroups

**Figure 6:**
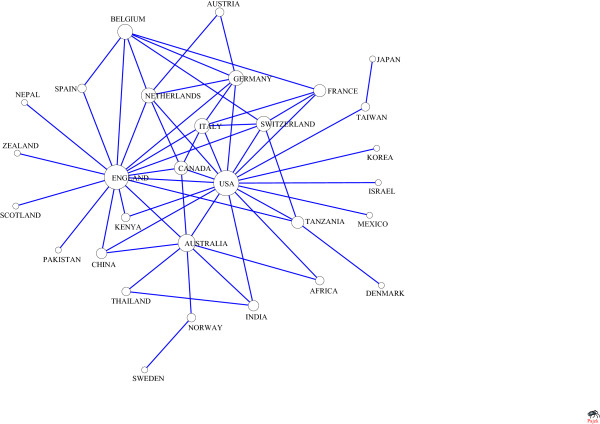
Collaboration among Countries/regions (line value>3).

The N-clique of countries/regions collaboration network in health management was shown in Table 7. The maximum is 9-clique. The M-core of multi-national collaboration network in health management was shown in Table 8. The maximum is 19-core. And the majorities are in 1-core, which contains 41 countries/regions. The collaboration network among the 30 countries/regions was shown in Figure 6. And we can find that USA, UK, and Australia are at the core of the map.

**Table 7:** The N-clique of countries/regions collaboration network in health management

**N-clique**	**Freq**	**Freq%**	**Representative**
0	17	16.67	SERBIA
1	10	9.80	CHILE
2	15	14.71	MEXICO
3	7	6.86	PAKISTAN
5	7	6.86	KOREA
6	10	9.80	BRAZIL
7	4	3.92	CHINA
8	10	9.80	CANADA
9	22	21.57	USA

**Table 8:** The M-core of multi-national collaboration network in health management

**m-core**	**Freq**	**Freq%**	**Representative**
0	18	17.4757	SERBIA
1	41	39.8058	FINLAND
2	14	13.5922	BRAZIL
3	7	6.7961	JAPAN
4	5	4.8544	TAIWAN
5	5	4.8544	SPAIN
6	3	2.9126	ITALY
8	2	1.9417	GERMANY
9	1	0.9709	SCOTLAND
11	3	2.9126	AUSTRALIA
17	1	0.9709	CHINA
19	3	2.9126	USA

### Analysis of hot research areas

#### Co-occurrence network

The keywords co-occurrence network contains 4356 nodes (keywords), 31628 lines (frequency of co-occurrence). The maximum frequency of co-occurrence is 97 and the network density is 0.0033345.

#### Analysis of co-occur network

**Figure 7:**
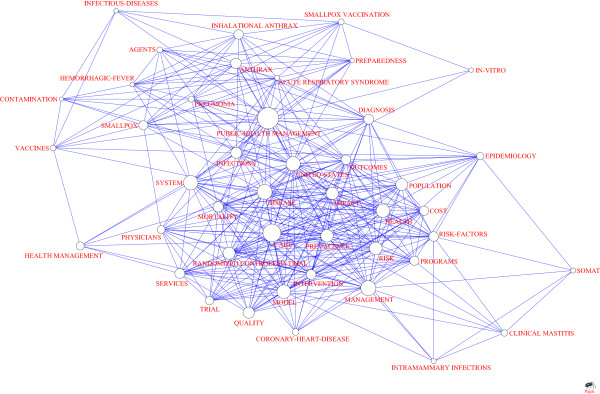
Keywords with co-occurrence frequency > 6.

As shown in Table 9, there are 44 keywords with frequency more than 20. Based on the m-core analysis, 44 keywords which is higher than 6-core were selected to form the network of Figure 7.

**Table 9:** 44 keywords with frequency more than 20

**Rank**	**Keyword**	**Frequency**
1	PUBLIC-HEALTH MANAGEMENT	171
2	CARE	120
3	MANAGEMENT	85
4	DISEASE	84
5	SYSTEM	79
6	UNITED-STATES	76
7	MODEL	71
8	HEALTH	68
9	RISK	61
10	PREVALENCE	59
11	IMPACT	57
12	RANDOMIZED CONTROLLED-TRIAL	55
13	POPULATION	53
14	QUALITY	48
15	INFECTIONS	46
16	CHILDREN	43
17	ANTHRAX	42
18	MORTALITY	40
19	PERFORMANCE	40
20	SERVICES	38
21	DIAGNOSIS	37
22	INTERVENTION	36
23	RISK-FACTORS	35
24	OUTCOMES	35
25	INHALATIONAL ANTHRAX	34
26	COST	33
27	ADULTS	33
28	SMALLPOX	32
29	PROGRAMS	32
30	WOMEN	30
31	PREVENTION	29
32	TRIAL	27
33	BEHAVIOR	26
34	HEALTH MANAGEMENT	25
35	HEALTH-CARE	25
36	QUALITY-OF-LIFE	24
37	TRANSMISSION	23
38	PHYSICIANS	23
39	RELIABILITY	22
40	PRIMARY-CARE	22
41	IDENTIFICATION	22
42	EPIDEMIOLOGY	21
43	DISEASE MANAGEMENT	20
44	COMMUNITY	20

The 44 keywords form a series of concentric circles with the most frequent sequence. Public Health Management, Care and Disease are placed in the center (Figure 7). Keywords in the innermost circle are those which have closest collaboration with Public Health Management. Keywords in the second innermost circle have closest collaboration with those which are in the innermost circle, and so on.

## Discussion

### The analysis of cooperation trend

Many studies have reported the ascending cooperation trend both in agencies and national cooperation. On the contrary, much less attention has been paid in the area of health management. As far as papers coauthored by author, there was a relatively high degree of cooperation in the field of health management research, between 1999 and 2011, 81% of output is the results of the research cooperation. Considering agencies and national cooperation level, however, the degree of cooperation is relatively lower, which has caused gaps by contrast with researcher cooperation level. Especially at the national level of cooperation, only 11% output is the results of international cooperation in the past 13 years, a little lower than 13% output in the Coronary Heart Disease research field in our previous study [43]. Therefore, the strengthening of international cooperation in health management research should be encouraged.

### The analysis of collaboration researchers

The maximum frequency of co-authorship is 8, which indicate that the collaboration in health management field is not tight comparing with other fields, such as oncology or cardiovascular field [44,45]. According to the centrality analysis, researchers such as O'Toole T1, Ascher Ms and Tonat K2 can be seen as the academic leaders in this field. According to the N-clique analysis and M-core analysis, the majority of researchers are in low N-clique and M-core, which once again proved that the research collaboration in health management research is not tight. The component analysis found that 22 research groups can be regarded as the backbone in this field. Therefore, the researchers in health management should strengthen their collaboration to improve the development and academic level of this field.

### The analysis of collaboration research institutions

Judging from the centrality, 37 research institutions such as UNIV MINNESOTA, USAF, UNIV WASHINGTON and UNIV MARYLAND play an important role in the information dissemination and resources control in health management. Similar to previous study in oncology or cardiovascular field [44-46], while in N-clique and M-core analysis, the frequency of 2- or 3-institutional collaboration is higher, which indicates that it is an irresistible trend that the scientific manpower of different institutions should be integrated. The 8 groups in Figure 5, formed by 33 institutions, co-published more than 3 times, could be regarded as the backbone in this field. It suggests that although to some extent there is collaboration among institutions in health management field, the level is not tight and stable. The government should encourage institutional collaboration to make their respective advantages complementary to each other, thereby, to further enhance the scientific research level. As depicted in Figure 5, extensive research collaboration existed in institutions of different types. For example, collaboration between university and hospital (CONCORD HOSP, UNIV WESTERN AUSTRALIA); collaboration between universities (UNIV FED RIO GRANDE DO SUL, UNIV SAO PAULO, UNIV FED SAO PAULO); collaboration among university, organization and government (WHO, CTR DIS CONTROL & PREVENT, JOHNS HOPKINS UNIV).

### The analysis of collaboration countries/regions

According to the N-clique and M-core analysis, international collaboration in health management is becoming an irresistible trend. Previous research showed that economic factor will improve the research collaboration [47,48]. And as shown in Figure 6, similar to previous study in oncology or cardiovascular field, those economic powers such as USA, UK are in the center of the network, which play an vital role in the information dissemination and resources control in health management. Although in developing countries/regions, such as China, research about health management started late, it has quickly becoming popular, and a broad collaboration network is forming. At the same time, other countries/regions which are less developed than China should also actively learn and cooperate with economic powers to enhance their scientific research level, change their position in information dissemination and control in this field, and to achieve the global balance development of health management.

### Analysis of hot research areas

To some degree, the frequency of keywords could reflect the hot research areas of health management around the world from 1999 to 2011, providing useful experience for researchers and policy makers. Normally, the highest frequency keywords tend to be the basic words of the field which are unable to be the reference of topic analysis. As the frequency of Public-Health Management and Care are far much higher than other keywords, they are basic words in this study. Besides, the keywords Management, Disease and System having higher frequency reflect the status quo of health management research as well. For instance, Disease shows that management of related diseases (especially Chronic diseases) and preventive care of high-risk groups are the key of health management research while system and model show that the constructions of health management system and model is one of the hot research topics. Risk, Prevalence, and Impact also indicates that researchers focus on the fields of health risk assessment and health management effect evaluation. Therefore, research of health management is mainly reflected in clinical medicine and preventive medicine, which refer to the groups of public, women and children. Research methods are concentrated in statistical analysis such as randomized controlled trials, etc. As for these high-frequency keywords involve cutting-edged issues, covering wide range, the higher scientific research productivity or cooperation of several institutes are one of the effective way to solve the problem.

### Limitations

This study using scientometrics methodology focuses mainly on the research collaboration among authors, institutions and countries/regions in Health Management research field with a view to some reference. With limited resources and research levels, this study only searched all the articles in the period 1999–2011 in Web of Knowledge, which content has certain limitations. Methods of SNA, co-authorship etc. are relatively fresh perspective but lacking innovation in this field. And lacking of regularity in the key word selection also impacts the analysis process. Besides, how is the research collaboration related with the research quality of the authors? What factors contribute to research collaboration? All of these need to be further investigated in future study.

## Conclusions

### Collaboration in health management field needs to be enhanced

The number of publications in the health management field is showing a rising trend, especially in recent years. Co-authorship is also keeping growing. And the cooperation of authors is obviously higher than that by institutions and countries. 22 research groups and 37 institutions devoted in this field, among which researchers or research team of USA and UK are in the core position in the collaboration network. Reviewing the related articles in other fields and comparing them with the research results in oncology or cardiovascular field in earlier stage, though 81% of the articles are produced by scientific cooperation, the Cooperation intensity in the field of health management is still relatively weak, especially between institutions and countries. Therefore, the important way of promoting the progress and internationalization of health management is to strengthen the cooperation between countries and institutions and take full advantage of the core role of dominant groups.

### Representative countries and authors are found in the networks

According to the centrality analysis, researchers such as O'Toole T1, Ascher Ms and Tonat K2 are representative to some extent in health management field. When it comes to institutions, network consisted of UNIV MARYLAND, USAF, CTR DIS CONTROL & PREVENT, WHO, MINIST HLTH is not only related to different types of institutions, but also shows the complicated relationship among them. Cooperating between countries/regions, USA and UK are in the center of the network which play leading roles in the information dissemination and resources control in health management. In order to provide a basis for the understanding of health management status quo and development trend, researchers should pay attention to the cooperation of representative scholars or institutions. They should also follow the cooperation model to study from the veteran organizations or institutions, take the opportunity of communication and cooperation, so as to promote the further research of their country or groups by using experience of countries or regions with developed health management research.

### The research topics in health management research

According to keywords analysis, in spite of its wider range, health management research relatively focuses on the clinical medicine and preventive medicine, which involve the groups of public, women and children. Research methods are also concentrated in statistical analysis such as randomized controlled trials, etc. In health management field of 1999–2011, the hot research topics, helping providing useful experience for researchers and policy makers, included that health risk warning of public, constructions of health management model and system, disease management and effect evaluation of health management, which provide useful basis for research direction.

In conclusion, by scientometrics methodology, this study analysing multiple collaboration types in Health Management research reveals the status quo, points out its defects and then comes up with related suggestions on strengthening cooperation of health management, analyzes the hot research topics as well. These results and proposal would provide an important reference for scholars, policy makers and managers on the aspect of researching and practicing health management deeply.

## Competing interests

The authors declare that they have no competing interests.

## Authors’ contributions

The work presented here was carried out in collaboration between all authors. CZ conceived of the study, participated in its design, drafted the manuscript. QY participated in study design, obtained data and contributed to interpretation, and helped draft the manuscript. QF performed the literature search and in the development of the manuscript. ZD provided the theoretical frameworks and performed much of the editing of the manuscript. All authors read and approved the final manuscript.

## Pre-publication history

The pre-publication history for this paper can be accessed here:

http://www.biomedcentral.com/1472-6947/13/52/prepub
